# Cardiodiagnostics Based on Photoplethysmographic Signals

**DOI:** 10.3390/diagnostics12020412

**Published:** 2022-02-05

**Authors:** Galya Georgieva-Tsaneva, Evgeniya Gospodinova, Krasimir Cheshmedzhiev

**Affiliations:** Institute of Robotics, Bulgarian Academy of Science, 1113 Sofia, Bulgaria; jenigospodinova@abv.bg (E.G.); cheshmedzhiev@gmail.com (K.C.)

**Keywords:** PPG signals, PPG device, heart rate variability, cardiovascular diseases, mathematical analysis, ROC analysis, syncope

## Abstract

The article presents a methodology to support the process of correct cardiodiagnostics based on cardio signals recorded with modern optical photoplethysmographic (PPG) sensor devices. An algorithm for preprocessing registered PPG signals and the formation of a time series for the analysis of heart rate variability is presented, which is an important information indicator in the diagnosis of cardiovascular diseases. In order to validate the proposed algorithm, an experimental scheme for synchronous recordings of PPG and electrocardiographic (ECG) signals and the study of the accuracy of the registered signals was created. The obtained results show high accuracy of the studied signals in terms of the following parameters: number of QRS complexes/pulse waves and mean RR intervals/PP intervals and the finding that the proposed algorithm is suitable for preprocessing PPG signals, as well as the possibility of interchangeable use of PPG and ECG. The results of the mathematical analysis of heart rate variability by applying linear methods (Time-Domain and Frequency-Domain) to two groups of people are presented: healthy controls and patients with cardiovascular disease (syncope). After determining the values of the parameters of the methods used, in order to distinguish healthy subjects from sick ones, statistical analysis was applied using *t*-test and Receiver Operating Characteristics (ROC) analysis. The obtained results show that the linear methods used are suitable for analysing the dynamics of PP interval series and for distinguishing healthy subjects from those with pathological diseases. The presented research and analyses can find applications in guaranteeing correctness and accuracy of conducting cardiodiagnostics in clinical practice.

## 1. Introduction

Diagnosis of cardiovascular disease can be assisted by mathematical methods for heart rate variability (HRV) [1,2] analysis based on the study of dynamic changes in cardiac activity. The dynamically changing heart rate is an indicator [3,4] of a person’s good health status, thanks to which his regulatory system adapts to changes in the external environment. Decreased HRV is considered a deterioration in the health of the individual and a reason to look for cardiovascular disorders [5] or vascular disease [6].

In recent years, there has been an increase in the use of HRV assessment due to the non-invasive nature of cardio signals, the clinical significance of the method, and the possibility of using it not only to study cardiovascular activity but also many other diseases (diabetes and psychopathological disorders) [7,8] and others.

Heart rate variability can be applied to a variety of cardiac signals: ECG signals, Holter records, and PPG signals. Remote patient follow-up [9,10] and the use of portable medical devices have gained popularity for monitoring heart rate, which does not require complex analyses of cardiac waveforms (ECG and PPG). The provision of preventive care and remote monitoring results in a reduction in the chronicity of diseases, which reduces mortality and contributes to the more efficient functioning of the health system.

Unobtrusive remote monitoring of the condition of patients at risk is an effective method to provide preventive care and maintain good health. Photoplethysmography is a relatively new technology that, thanks to the development of technology and miniaturization, can be used for continuous or periodic observation of individuals.

Photoplethysmography makes it possible to measure various parameters that take into account the health of the human body, such as heart rate, blood pressure, oxygen saturation, and others. PPG sensor technology [11,12] is a non-invasive, easily accessible method for detecting changes in blood volume in arterial blood vessels by using optical methods. Blood flow has a pulsating character and, therefore, the volume of blood changes [13,14] and can be measured in certain places of the human body through changes in the intensity of light when reflected or passing through the tissue. These differences reflect changes in blood perfusion of human tissue.

The PPG sensor consists of an infrared emitter that transmits light through the skin to the blood vessels and a detector that responds to the light transmitted or reflected by the blood vessels. In most cases, the emitter and detector are located in a small device placed on a finger or toe, earlobe, wrist, and forearm [15]. 

The shape of the recorded signal indicates fluctuations in blood volume and, therefore, reflects the heart rate [16,17]. The light read has a variable component (called an AC component) and a quasi-DC component. Changes in the AC component are due to changes in arterial blood volume. The changes in the DC component are due to the following factors: average blood volume, optical properties of the skin, respiration, vasomotor activity.

Mathematically based analyses of the form of PPG signals provide useful clinical information on several medical issues concerning the general health of the human body such as blood pressure, respiratory processes, sympathetic nervous system activity, the study of heart rate variability, detection of irregular heartbeat, infectious diseases, diabetes, and others.

Heart rate variability can be determined based on the detected P peaks in the PPG signal and the PP intervals time series formed on their basis. HRV in PPG signals determines the variation of the time series formed by the PP intervals and corresponds to the variation of the RR intervals in ECG signals.

Heart rate variability, determined based on the use of PPG signals, is a relatively little studied method, and at the same time, PPG sensors are convincingly entering the everyday life of modern people. The authors of [18] studied PPG records of patients diagnosed with diabetes using HRV parameters in the time and frequency range. Publication [19] examined healthy young and adult individuals using HRV analysis of PPG signals. The influence of psychological factors on HRV was studied in a publication [20] based on PPG and ECG signals. 

One of the aims of this article is to study the HRV parameters of PPG signals and compare them with HRV parameters obtained from simultaneously recorded ECG signals.

Two types of cardio signals (PPG and ECG) were used in this study to compare the heart rate variability parameters of patients diagnosed with syncope and a healthy control group. Syncope (according to the European Society of Cardiology) is a transient loss of consciousness resulting from hypoperfusion of the central nervous system, and it occurs suddenly; the condition is short-lived, and then it spontaneously restores the normal state of the organism [21]. Syncope is a serious individual condition that is most often caused by stress, can rarely be predicted, and is the cause of additional tension in people who have experienced it. The condition does not require additional medical interventions [22,23] but is a common reason to visit the emergency department or call an ambulance at home. For this reason, syncope cases place an additional burden on health services [24] and are a source of considerable costs for the health system.

### 1.1. Background

Research in the scientific literature shows the possibility of photoplethysmographic signals to be extracted, processed, and analyzed and have conclusions drawn to be applied in healthcare and medicine to support diagnosis and adequate treatment. 

The authors of [25] present a portable device using an integrated photoplethysmographic optical sensor to monitor heart rate and several built-in sensors to monitor the physiological sleep of users, distinguishing between the phases of deep sleep and regular sleep. The device was tested on volunteers during a seven-day collection of photoplethysmographic signal records.

Publication [26] explores the possibility of obtaining PPG data in people’s daily lives. A volunteer was asked to wear a photoplethysmographic wrist sensor device 6 days a week, 4 weeks, and to record the activities being performed. The research performed aims to study the quality of the recorded signal. The analyses performed show good quality of the recorded signal during 30.3% of the time during which the device was worn. Research shows two things: the ability to obtain information by using PPG sensors, and it also shows the need to improve this process.

In [27], the authors used multi-site photoplethysmography (MPPG) to record pulse wave (index finger, toe, and ear) and heart rate analyses to examine arterial stiffness in pediatric patients after a successful heart transplant. The peripheral heart rate is recorded and the arrival times from the different heart rate readings are compared.

The authors of the publication [28] investigated the influence of the signal registration site by studying the differences in the form of signals registered on the finger, toe, and forehead. Pulse amplitude variation in the photoplethysmographic signal has been studied and shown to be an alternative to pulse pressure variation in the arterial blood pressure signal. The study was performed on twenty-nine patients who had recently undergone surgery.

The authors of [29] studied PPG signals recorded from six different locations in 36 healthy individuals (finger, lower wrist, upper wrist, forehead, and ear), studying the influence of breathing type (normal and deep) on the shape of the pulse wave and its characteristics. The authors observed changes in some of the signals recorded during deep breathing, due to which they cannot be used to extract the characteristics of the waveform.

In [30], non-invasive estimation of glycated hemoglobin and SpO_2_ was performed using a portable device based on Multi-Wavelength PPG. Thus, the proposed method makes it possible to monitor blood sugar levels and diagnose diabetes at an early stage of the disease.

The authors of [31] consider the use of photoplethysmographic optical sensor to examine the cardiovascular system from different parts of the surface of human skin. Transmission-type PPG sensors placed on the fingers and wrist were tested. The authors studied the conditions, limitations, and recommendations for the development of a wearable PPG sensor device operating in a magnetic field.

Publication [32] discusses the use of PPG devices for remote patient monitoring. The various sources of noise (individual variation, physiological processes, and external perturbation) that were obtained when recording cardiac data using PPG sensors were evaluated. 

The authors of the article present an initial version of an integrated photoplethysmographic-based system for cardiovascular monitoring in a publication [33]. The presented system consists of a portable PPG device for PPG signals recording and a software system with server architecture for processing, storing, and analyzing input data. The studies were performed on patients diagnosed with cardiovascular disease and healthy controls. Mathematically based analysis of cardiological data in the time and frequency domain with nonlinear methods was performed.

The accuracy of recording photoplethysmographic signals depends on the place of registration on the human body. The authors of publications [34,35] report that the signal quality and the shape of the pulse wave depend on the location of the sensor. For example, there is not much tissue in the wrist area, but there are many bones, tendons, and muscles that can cause a decrease in the efficiency of recording signals from optical sensors. The operation of optical sensors is also influenced by ambient temperature, for example, at low outdoor temperatures reduce blood flow. At the same time, the very rapid passage of blood flow (for example in fast heart rate) in the field of measurement also results in a slight reduction in the accuracy of registration. 

PPG signals, similarly to all biomedical signals, contain noise components that must be optimally reduced before applying mathematical analysis to the studied data. This process is very important from the point of view of achieving reliable results in the study of heart rate variability. The sources of noise [36] can be individual characteristics of the patient (skin thickness, age, sex, obesity, etc.), physiological processes (respiration, temperature, location of the sensor, and venous pulsation), and external disturbances caused by movement, environment, and outdoor light). 

PPG signals can be used for prognostic and diagnostic purposes, but a necessary condition is that the shape of the PPG signal is not distorted by noise. For this reason, it is essential to include filtering and detrending at the preprocessing stage [37].

### 1.2. The Purpose of This Article

The complexity of the organization of the cardiovascular system and the mechanisms of its regulation results in an increased interest of researchers in this topic, while there are still many unresolved issues related to preprocessor processing of photoplethysmographic signals and their applications in the diagnosis and prognosis of cardiovascular disease.

The main objectives of this article are as follows:

- Presentation of an algorithm for preprocessing of registered PPG signals via a portable device;
- Presentation of an experimental scheme for synchronous recording of ECG and PPG signals and comparative analysis of the determined PP and RR interval series in terms of the accuracy parameter;
- Research by conducting mathematical analysis (linear) of two types of cardio signals: ECG and PPG and analysis of the possibility of equality of the two types of examined signals for diagnostic purposes;
- Analysis of the ability to distinguish between healthy individuals and patients with cardiovascular syncope by applying linear methods that are standardized for analysis and assessment of heart rate variability.

## 2. Materials and Methods

The creation of modern diagnostic equipment is a priority in the development of medicine and healthcare. Tracking changes in the parameters of the physiological systems of the human body by using modern technical means opens up opportunities for improving therapeutic and diagnostic methods in medical practice in critical conditions. 

The developed cardiology system performs collection and processing of photoplethysmographic data, analysis of the received information, determination of diagnostic indicators to present the results in an easy-to-understand manner by doctors and patients form. Data collection is based on the registration of PPG signals, reflecting the functioning of the cardiovascular system in a form convenient for further processing, analysis, and evaluation of the obtained data. This information is important for diagnosing the condition of the patients’ cardiovascular system and is an additional criterion for assessing cardiovascular risks, as well as for determining the effectiveness of the applied treatment.

The experimental cardio system by which the photoplethysmographic and electrocardiographic signals were obtained in this study consists of a device for recording PPG signals and software for pre-processing and analysis of signals.

### 2.1. Device for Recording PPG Signals 

The developed device is designed for the autonomous study of the health status of a wide range of users. The interest in this type of medical devices is due to the possibility of continuously monitoring patients at work and at home.

The device operates in the following two modes:

- Passing light through fabric using a discrete sensor that is external to the device and is in the form of a clip, which is placed on the finger or earlobe;
- Reflection of light from the human tissue through an integrated sensor built into the device itself.

In the reflection mode, the light source and the photodetector are placed on the same side of the human tissue, while in the second mode, it is placed on both different sides of the tissue.

**Figure 1:**
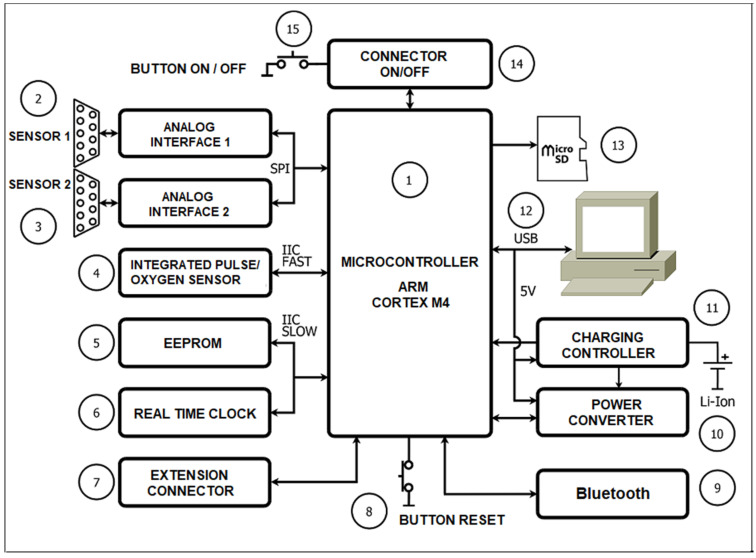
Block diagram of a device for recording PPG signals.

Figure 1 shows a diagram of the experimental system for recording photoplethysmographic signals, their conversion into digital data, and their processing. The system consists of the following main components:

1. Microcontroller;

2 and 3. Analog circuit for connection of external discrete sensors;

4. Integrated heart rate and oxygen sensor;

5. EEPROM;

6. Real Time Clock (RTC);

7. Expansion connector;

8. RESET button;

9. Wireless communication module (Bluetooth);

10. Power supply and control scheme;

11. Scheme for charging the accumulator battery;

12. Connector for USB interface;

13. Memory card connector;

14. On/Off controller;

15. On/Off button.

The advantages of the developed device include the following:

- The high sensitivity of measurements;
- Ability to work with two types of sensors: discrete in the form of a clip and integrated into the device;
- Portable dimensions: 95 mm/20 mm and low weight: 120;
- Easy to use;
- Possibility stably positioning the device at different parts of the body: finger, ear, etc.;
- The disadvantages of this device include the following:
- Influence of various artifacts as a result of the movement of the studied individual;
- Dependence of research on the condition of the surface of the biological tissue of the individual, as the research may not be accurate in the case where the patient has rougher skin.

The portable device for recording PPG signals created by the authors has been described in a previous publication [33] and is not subject to presentation and analysis in this article.

### 2.2. Algorithm for Preprocessing of PPG Signals

**Figure 2:**
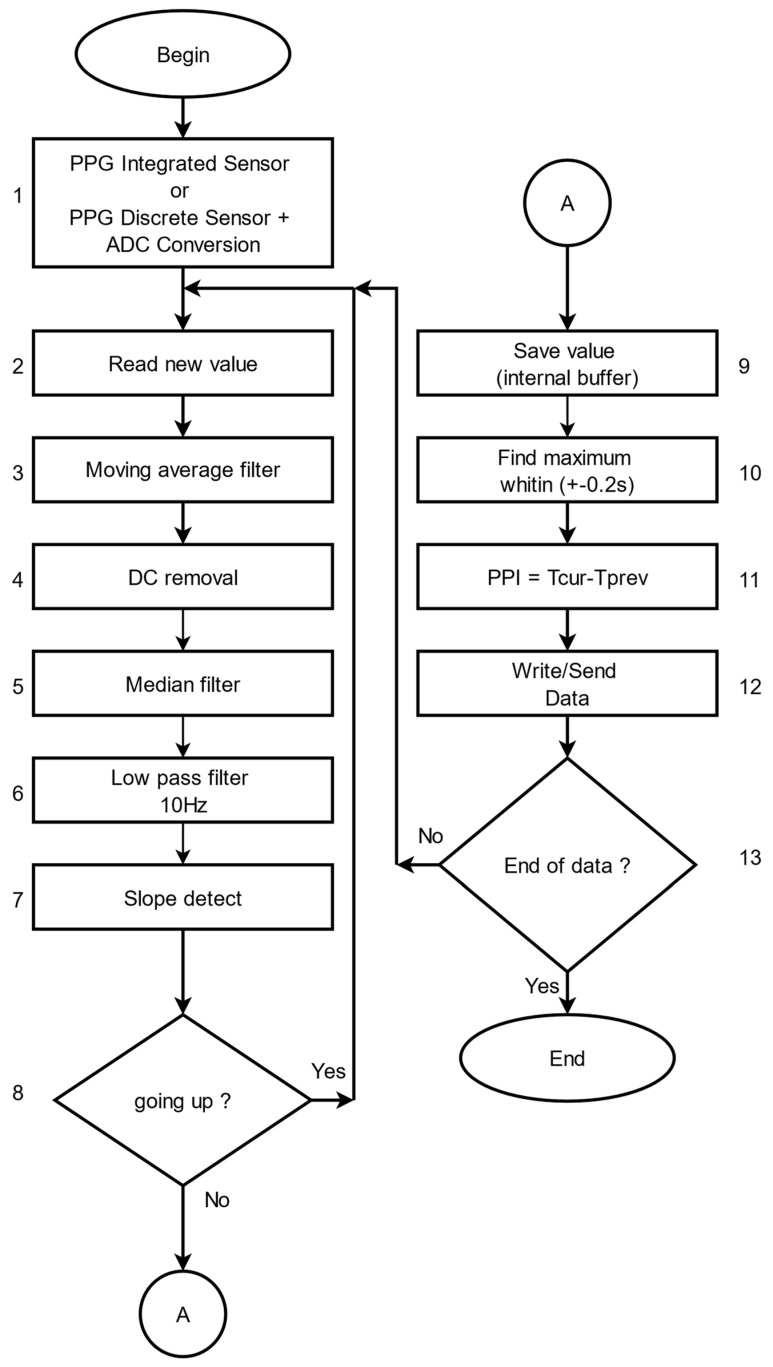
Algorithm for determining PP interval series of the photoplethysmographic signal.

**Figure 3:**
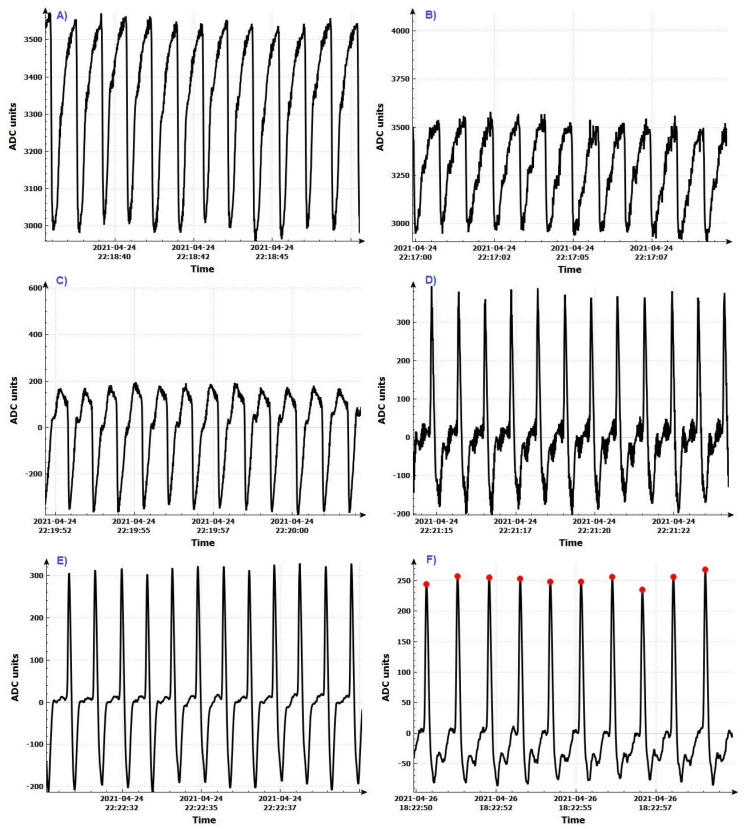
Conversion of the signal format after the individual procedures in pre-processing. (**A**) After applying analog-to-digital conversion; (**B**) after applying an averaging filter; (**C**) after removing the DC component; (**D**) after applying a median filter; (**E**) after applying a low-pass filter; (**F**) determination of P vertices.

The software for preprocessing PPG signals, which have been recorded with the presented device, is written using C++ language. The block diagram of the algorithm for preprocessing PPG signals is shown in Figure 2. This algorithm is used to eliminate noise in the signals and to obtain the PP interval series, based on which analysis and assessment of patients’ health can be performed. The main steps of the algorithm are as follows:

Step 1: Register the PPG signal by using an integrated digital sensor or a discrete sensor after analog-to-digital conversion. The signal shape after analog-to-digital conversion is shown in Figure 3A.

Step 2: Read the value from the digital signal that becomes current.

Step 3: An averaging filter is applied to eliminate small, short-term disturbances in the signal. The averaging filter works as follows:

Addition of the current read value to the previous value;

- Verification that the number of reading values has reached the present number. If the present number is reached, the total number is divided by the number of reading values and the arithmetic mean value thus obtained is used for further signal processing. Otherwise, the next value of the signal is read.

After applying this step of the algorithm, the signal has the form shown in Figure 3B.

Step 4: The constant component of the signal is removed (Figure 3C). The signal received from the sensors contains two components: constant and variable. Only the variable part of the signal is needed to determine the P vertices. The following digital filter was used to remove the DC part:

(1) $$y_{n}=x_{n}-x_{n-1}+R_{y_{n-1}},$$

where

*x_n_*—input value;
*y_n_*—output value;
*R*—parameter accepting values from 0.9 to 1; in this case, *R* is 0.95.

Step 5: Eliminate the large deviations in the input data by applying a median filter (Figure 3D). The principle of the operation of this filter is as follows:

1. The last n values of the signal are taken, which are sorted by size;
2. If n is an odd number, the middle element is selected;
3. If n is an even number, the arithmetic mean of the two mean elements is calculated.

Step 6: Remove the high frequency components of the input signal by applying a low pass filter (Figure 3E). If the maximum heart rate is 220 beats/min, then the heart rate is about 3.7 Hz. Each signal with a higher frequency must be suppressed so as not to affect further signal processing. A digital low-pass filter with an upper cut-off frequency of 10 Hz is used:

(2) $$y_{n}=k_{1}(x_{n}+x_{n-1})+k_{2}y_{n-1},$$

where

1. k1—filter coefficient calculated for sampling frequency 100 Hz;
2. k2—filter coefficient calculated for the upper cut-off frequency 10 Hz.

Step 7: Determine the slope of the signal curve as follows: If the current value is greater than the previous value, then the maximum value is updated and the process continues to process the next incoming data.

Step 8: Determine the P peak as follows: If the following condition is met and the current value is less than the previous signal value, this means that the curve has a downward direction and this is the P peak. Otherwise, read the next value of the signal.

Step 9: Remember the current position of the maximum value in an internal buffer of 0.2 s (corresponding to a different number of values depending on the sampling frequency).

Step 10: A second check is made to observe if the maximum is not incorrectly determined by searching again the data for the last 0.2 s to determine the exact position of the P peak. The signal with certain P peaks is shown in Figure 3F.

Step 11: Determine the distance (time) between two consecutive P peaks, as the time positions for each signal peak are known. The distance between two consecutive P peaks corresponds to the time between every two consecutive heartbeats and is determined by the following formula:

PPI[s] = Tn − Tn−1,(3)

where

1. PPI—Pulse to Pulse Interval—the time between two adjacent intervals;
2. Tn—the time of the n peak;
3. Tn−1—the time of the n−1 peak.

Step 12: Record the received data (PP interval series) in the local memory of the PPG device, or data are sent to a computer for mathematical analysis to diagnose cardiovascular disease.

Step 13: Check for end of data. If there are no more data to process, the algorithm ends, otherwise, the algorithm proceeds to step 2 of the algorithm.

### 2.3. Verification of the Algorithm for Determining the PP Interval Series

In order to verify the proposed algorithm for determining the PP interval series, an experimental setup was created for the simultaneous (synchronous) recording of ECG and PPG signals of healthy individuals and a comparative analysis of the registered signals in terms of the accuracy parameter.

**Figure 4:**
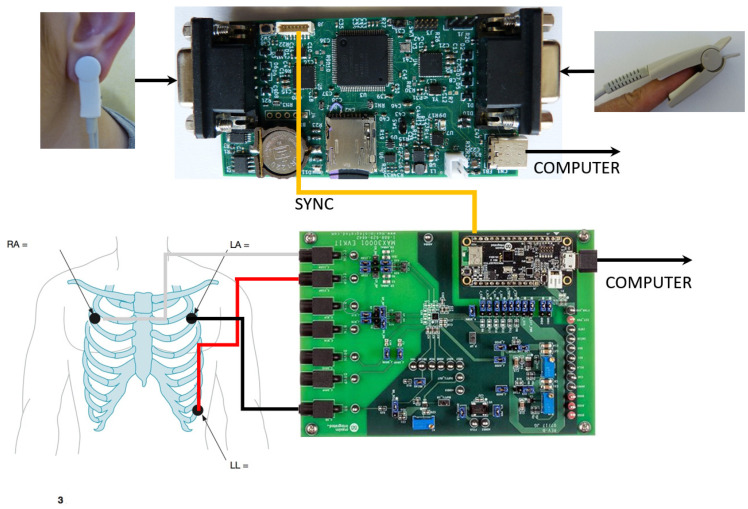
Experimental setting for simultaneous recording of ECG and PPG signals.

Figure 4 shows the experimental setup, which consists of the following several main blocks:

- ECG signal recording system;
- PPG device with two sensors placed on the patient’s finger and ear for recording PPG signals.

The development system was used to record electrocardiographic signals (MAX30001EVSYS) [38]. A software procedure has been created for the development system to enable synchronization with a PPG device. 

The data received from these sensors are sent to a personal computer for further processing, including obtaining PP interval series from the ear and finger of the subject.

A comparative analysis between the registered ECG and PPG signals was made using software created by the authors. The following data from two groups of people were used for comparative analysis: healthy subjects and patients with syncope:

- RR intervals received from the ECG sensor, calculated by the software of the development system;
- PP intervals obtained from the PPG sensor placed on the patient’s finger, determined by the created software;
- PP intervals obtained from the PPG sensor placed on the patient’s ear, determined by the created software.

The created experimental system for recording ECG and PPG signals can connect to a personal computer via a USB interface. In offline mode, the system records RR/PP interval series, which are then analyzed.

## 3. Results

### 3.1. Comparative Analysis between ECG and PPG Signals in Terms of Parameter Accuracy

The comparative analysis of the registered ECG and PPG signals with the presented experimental scheme was performed concerning parameter accuracy by determining the root mean square error and the relative error on the following two groups of people:

Healthy individuals;
Patients diagnosed with syncope.

The demographic characteristics of the studied records of healthy individuals and patients diagnosed by a cardiologist with syncope are presented in Table 1. The table provides information on age (average age of individuals) and provides a gender distribution. In the 46 syncope records analyzed, patients were aged 32–58 years, including 20 men and 26 women. The healthy records analyzed were 48 persons aged 30–52 years inclusive, of which 21 were men and 27 were women. Values are expressed as mean ± standard deviation (sd) or in percentages (%). There is no significant difference between the different groups according to their demographic characteristics. Research was conducted on previously anonymized records.

In order to demonstrate the coincidence of the intervals between the two types of studied signals, the mean square error (MSE) was determined by the following formula:

(4) $$MSE=1/N\sqrt{\sum_{i=1}^{N}{\left(x_{i}-y_{i}\right)}^{2}}\;,$$

where *x_i_* denotes QRS or mean RR intervals;

*y_i_* denotes pulse waves or PP intervals;
*N* denotes the number of intervals.

**Figure 5:**
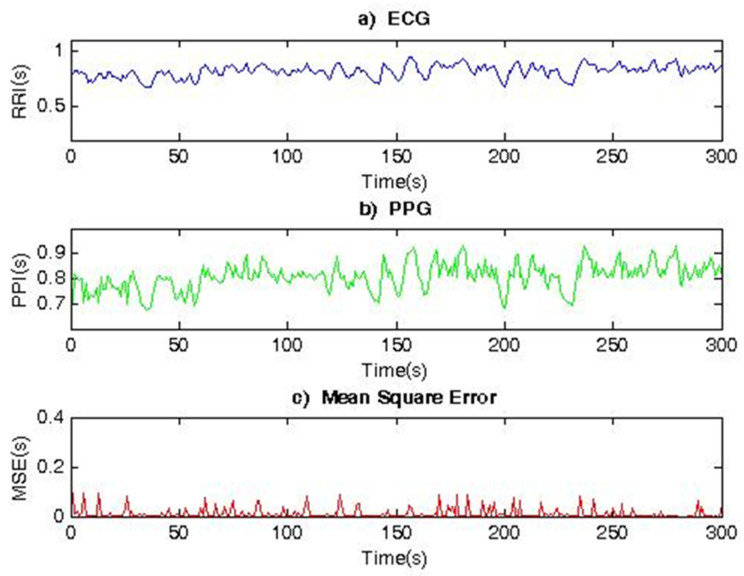
Comparison of RR and PP interval series for a healthy individual.

Figure 5 shows two 300 s segments corresponding to the RR and PP interval series, registered by an ECG and PPG sensor placed on the finger of the hand, as well as their Mean Square Error (MSE = 0.0242). The data refer to a healthy subject. The determined MSE is very small, which indicates that the two signals taken by electrocardiography and photoplethysmography provide identical heart rate variability series. This shows that, for healthy individuals, RR and PP interval series (HRV series) have similar shapes and characteristics and can, therefore, be used interchangeably or in parallel to study the cardiovascular system of individuals.

**Figure 6:**
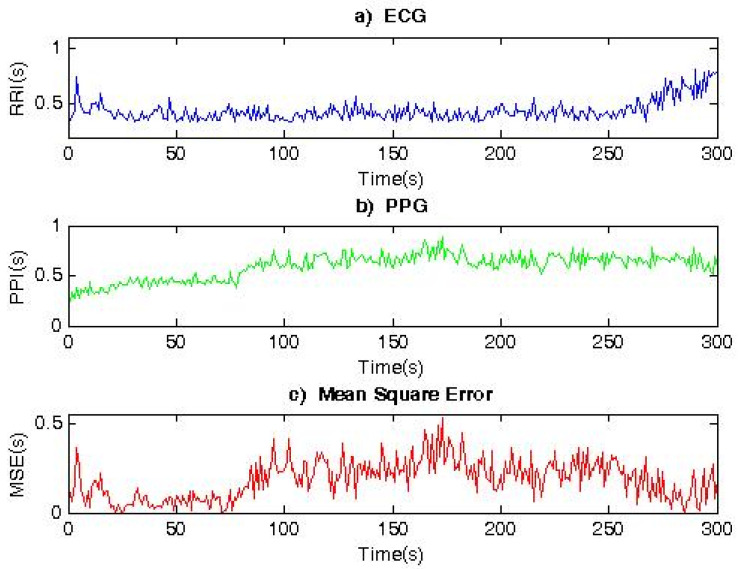
Comparison of RR and PP interval series for a syncope patient.

Figure 6 shows the RR and PP interval series, as well as the MSE (MSE = 0.235), for a patient with syncope. The obtained average value for the MSE parameter is 10-times higher than in a healthy individual. The obtained graphs (Figure 6) show differences in the form of an HRV time series.

Table 2 shows the results for healthy individuals and Table 3 shows the results for patients with syncope. The following parameters were studied:

- Number of QRS complexes and number of pulse waves of the three types of signals: ECG, PPG1 (recorded with a sensor placed on the finger), and PPG2 (recorded with a sensor placed on the ear);
- The average value of RR intervals / PP intervals (ms);
- The relative errors ECG-PPG1, ECG-PPG2.

Based on the obtained results, the following conclusions can be made:

- The relative error between the studied parameters in healthy individuals (for the studied ECG and PPG signals) is very small; therefore, the proposed algorithm for determining PP interval series (for PPG) is suitable and can be used for preprocessor processing of PPG signals and determination of PP series;
- The relative error between the studied parameters for patients with syncope (for the studied ECG and PPG signals) is greater than 15% because synchronization between ECG and PPG signals is disturbed. The synchronization between the two types of signals is disturbed due to pathological changes in the cardiovascular system of the studied patients. This information is important for the diagnosis of patients and is an additional criterion for assessing the risk of cardiovascular disease. The presence or absence of synchronization between PPG and ECG is an important indicator in determining the effectiveness of treatment of patients with such diseases.

### 3.2. Comparative Analysis between ECG and PPG by APPLYING Linear Methods for HRV Analysis

The comparative analysis between ECG and PPG signals (recorded using a PPG sensor placed on the index finger of the hand) by applying linear methods was performed on two studied groups: healthy control group (mean age 44 years) and sick individuals diagnosed with syncope by a cardiologist (mean age 43 years). Statistical significance between the studied groups was determined by applying a statistical *t*-test. Table 4 shows the results by applying linear methods of analysis: analysis in the time and frequency domain.

The results obtained in the time domain (Table 4) show that the values of parameters SDNN, SDANN, and RMSSD are significant (*p* value < 0.05) in the comparison of cardio records of the healthy control group and patients diagnosed with syncope. The values of these parameters are significantly lower in patients with syncope compared to healthy individuals. In PPG, the value of SDNN is 84.07 ± 22.03 ms (versus 143.41 ± 39.48 ms in healthy); SDANN was reduced to 89.14 ± 49.56 ms (versus 146.07 ± 43.51 ms in healthy subjects); and RMSSD decreased to 22.04 ± 19.11 ms (versus 38.03 ± 12.09 ms in healthy subjects). Therefore, the statistical parameters SDNN, SDANN, and RMSSD can be used to distinguish sick individuals from healthy ones.

The analysis of HRV through the application of geometric methods consists in determining the geometric shape of the distribution of RR/PP intervals and their derived geometric figures. In this case, the following two approaches are used in the analysis of the RR/PP interval series:

1. Construction of a histogram consisting of normal RR/PP intervals and its triangular approximation by defining the following two parameters: HRV triangular index and TINN;

2. Construction of a scattergram (Poincare plot) in which the values of two adjacent RR/PP intervals are plotted on the axes of the rectangular coordinate system.

**Figure 7:**
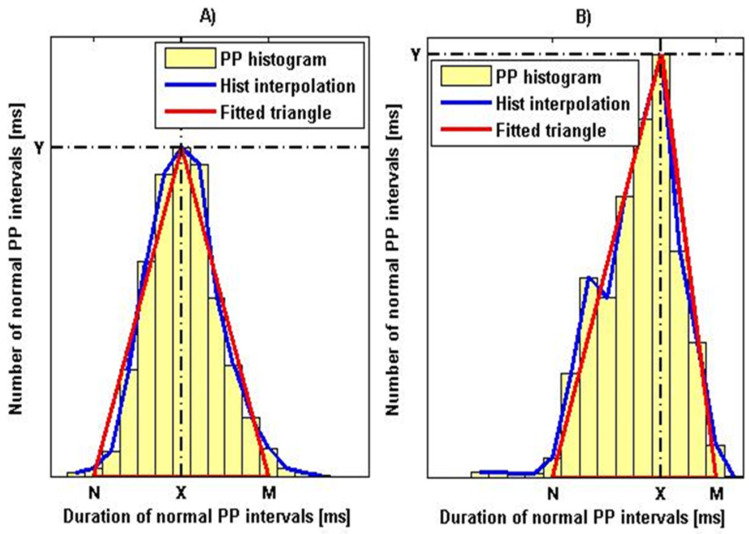
Histogram of PP interval series (**A**) for a healthy individual; (**B**) for a patient with syncope.

Figure 7 shows histograms with a triangular interpolation of the PPG signal, as Figure 7A shows the histogram of a healthy individual with parameter values HRVi = 18.7 and TINN = 294,500 ms. Figure 7B shows the histogram of a patient with syncope with parameters: HRVi = 8.7 and TINN = 537,500 ms. Similar graphs were obtained for the ECG signals of the two types of patients studied.

**Figure 8:**
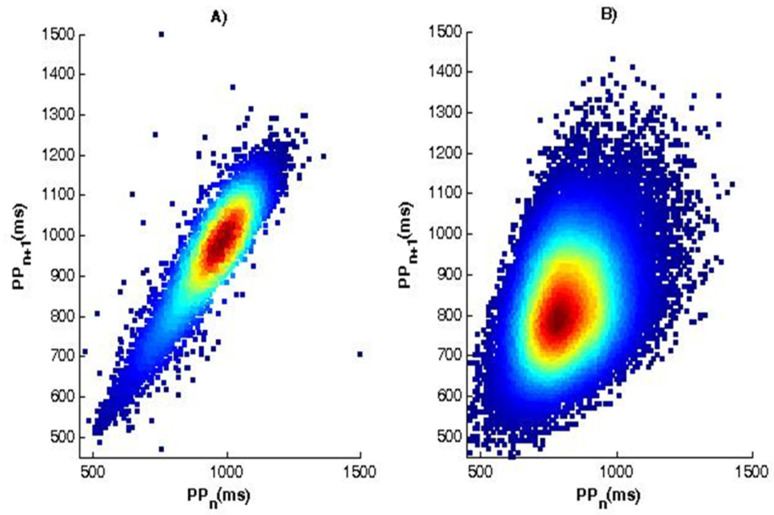
Scaterogram of PP interval series (**A**) for a healthy individual; (**B**) for a patient with syncope.

The essence of the method of scattergram (correlation rhythmography) consists in the graphical construction of consecutive pairs of cardio intervals in the form of points, and the value of the current interval is plotted on the abscissa RR/PP (RRn/PPn) and on the ordinate—the value of the next interval RR/PP (RRn+1/PPn+1). The set of points thus obtained forms an autocorrelation cloud. Cardiac arrhythmias can be determined by the type of cloud, as large deviations in the duration of RR/PP cardio intervals from the mean value result in significant scattering of points in the plane (RR/PPn, RRn+1/PPn+1). The normal shape (Figure 8A) of the scattergram is in the form of a comet with a pointed lower part, which expands in its upper part. In a patient with syncope (Figure 8B), the scattergram has the shape of a torpedo.

For the frequency domain, the results presented in Table 4 show high Power VLF values for both healthy and sick individuals, and the differences found are not significant (*p* value > 0.05).

The obtained results show a lower relative share of frequencies in the LF band for the power of the signal in the indicators of patients diagnosed with syncope compared to the values of healthy individuals (818.84 ± 232.06 ms^2^ versus 1873.35 ± 406.25 ms^2^, the ECG). The low frequency LF range reflects the influence of the sympathetic nervous system. The results show the lower activity of the sympathetic nervous system in patients diagnosed with syncope. At the same time, an increase in the relative share of the signal power in the high-frequency range is reported (1297.22 ± 341.34 ms^2^ versus 1110.42 ± 511.57 ms^2^ the ECG), which has no significant significance because *p* value > 0.05.

The significant reduction in energy in the LF range is reflected in the LF/HF ratio, which decreases significantly and the values obtained (0.63 ± 0.41 versus 1.69 ± 0.76) are significantly below normal for healthy people (recommended in the Standard for Heart Rate Variability [39]). 

### 3.3. Statistical Evaluation of Diagnostic Differentiation of PPG Signals by Using Receiver Operating Characteristic Analysis

In the present article, PPG signals were studied in order to distinguish healthy subjects from patients with syncope, and they were applied in addition to the *t*-test method for determining p-value and Receiver Operating Characteristic (ROC) analysis. ROC analysis is a graphical method for presenting the results of the binary classification and evaluating the effectiveness of the classification. The method is based on the construction of ROC curves, which do not work with absolute indicators for the correct classification of results but with relative indicators:

- Sensitivity—the proportion of positive cases that have been correctly classified by the model;
- Specificity—the share of negative cases that have been correctly classified by the model.

**Figure 9:**
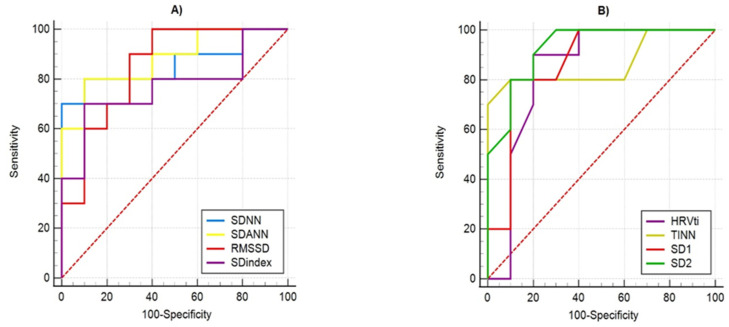
ROC curves of parameters for Time-Domain analysis of PPG data for differentiation healthy from unhealthy (with syncope) subjects: (**A**) by statistical parameters; (**B**) by geometrical parameters.

**Figure 10:**
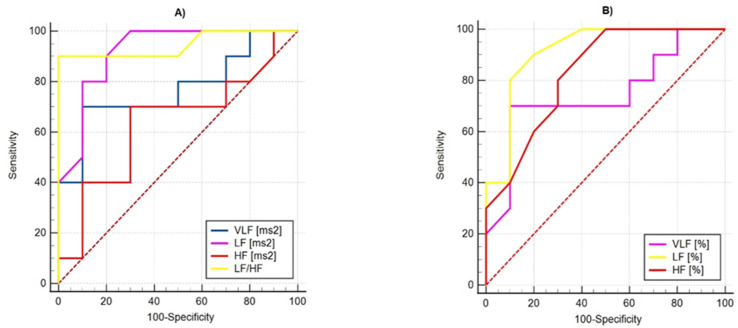
ROC curves of parameters for Frequency-Domain analysis of PPG data for differentiation healthy from unhealthy (with syncope) subjects: (**A**) by parameters in ms^2^; (**B**) by parameters in %.

The ROC curves of the studied parameters by determining their sensitivity and specificity are demonstrated in Figure 9 and Figure 10. The visual evaluation of ROC curves is not sufficient to analyze the parameters of the linear methods used: Time-Domain and Frequency-Domain analysis. A specific method to compare ROC curves is to estimate the area under the curves (AUC), which is a measure of how well a parameter can distinguish between two diagnostic groups. When there is a perfect separation of the values of the parameters of the two groups, the area under the ROC curve is equal to 1.0. Otherwise, when the parameter cannot distinguish between the two groups, AUC will be equal to 0.5. Table 5 shows the AUC values of the studied parameters, which show that they can distinguish the two groups of subjects, and their quality varies from good (SDindex, Power VLF (%)) to excellent (SD2, Power LF (%), LF/HF ratio).

## 4. Discussion

The studies performed by the authors revealed large values of the relative error in determining the number of QRS complexes and the average value of ECG and PPG time intervals. In PPG1 (a sensor on the finger) and PPG2 (a sensor on the ear) in patients with syncope, the relative error is greater than 15% (Number of QRS complexes/pulse waves) and over 17.5% (Mean RR intervals/PP intervals). This indicates that the synchronization between ECG and PPG signals is disturbed during the recording. The reason for the violation of the synchronization between the two types of signals may be long-term pathological changes in the cardiovascular system of the studied patients diagnosed with syncope. Studies have shown that the relative error in determining the number of QRS complexes and the average value of ECG and PPG time intervals can be used as an indicator of the presence of cardiovascular disorders in the studied individuals.

Advances in semiconductor and optical technology have resulted in the improvement of photoplethysmography and the expansion of its use in people’s daily lives, including monitoring patients suffering from cardiovascular disease. PPG sensors become more compact and easier to install in portable devices for remote continuous monitoring of heart rates. The characteristics derived from PPG and ECG signals are a good tool for application in early diagnosis and taking preventive measures to maintain human health. 

Thanks to new technologies that reduce the size and weight of PPG sensors and devices based on them, the comfort of using PPG devices for monitoring, prevention, and accurate diagnosis of individuals increased. The quality of the cardio signals obtained by photoplethysmography is improved, and the results obtained by the evaluation of HRV on PPG signals are accurate enough to make correct diagnostic decisions.

The paper presents the application of advanced PPG sensors in portable devices for remotely monitoring cardiac activity. The presented algorithm for determining the P peaks of PPG signals is programmatically implemented and tested in a PPG device created by the authors. The received PPG signals are processed and analyzed, and the results (numerical and graphical) are stored in a computer. The authors consider the idea of creating an information platform in which it is possible to store the results of mathematically based analyses of the studied individuals’ cardio.

The obtained low values of the MSE parameter show that the signals taken by electrocardiography and photoplethysmography have identical HRV series in healthy subjects. The analyses conducted made it possible to confirm that, in cardiac studies, one can choose between PPG and ECG depending on the specific real situation in terms of which signal for the individual is easier or more convenient to record.

The studies in this article were performed on real cardiac records (two types of data were taken: ECG and PPG) of 46 patients diagnosed with syncope by a cardiologist, as well as on 48 healthy volunteers. This improves the value of the research conducted and makes the results of this research important for research activity.

Studies show that the statistical parameters SDNN, SDANN, and RMSSD have significant significance (*p* value < 0.05 when performing a *t*-test). These parameters have lower values in patients diagnosed with syncope, which is an indicator of reduced heart rate variability.

The obtained histograms with triangular interpolation of the PP interval series of the studied healthy and diseased individuals show the dependence of the basis of the histogram on the variability of heart rate (a wider base corresponds to a greater variability in heart rate and vice versa; narrow base indicates the possibility of a pathological cardiovascular condition). 

The advantage of the geometrical parameters HRVti and TINN is that they allow not taking into account PP/RR intervals, which are associated with artifacts and extrasystoles and which would significantly distort the actual picture in the analysis of HRV. Geometric methods can serve as an alternative to statistical methods provided that the duration of the recordings is not less than 20 min.

The application of correlation rhythmography in the studied two types of ECG and PPG signals shows that the geometric shape of the depicted points (depending on the scattering of points in the plane) can be inferred from the heart rate of the individual. The normal shape of the scattergram has the shape of a comet with a pointed lower part, and the other shapes carry information about heart disease. 

Studies in the frequency domain show that the strength of the studied signals in the VLF domain is (*p* value > 0.05) in the definition of the two studied groups (healthy and diseased). In the LF area, the signal Power in patients diagnosed with syncope is significantly lower than in the control healthy group, which indicates a decrease in the activity of the sympathetic nervous system.

A significant reduction in signal energy in the LF range contributes to a reduction in the sympathovagal balance index (LF/HF) in patients diagnosed with syncope below normal for healthy individuals. 

## 5. Limitations

This study has several limitations. The studies were performed with a small number of ECG and PPG signals (46 records of persons diagnosed with syncope and 48 records of participants in the healthy control group). The authors are working on building an extensive database of cardio records of people with various cardiovascular diseases. The ECG and PPG recordings of patients with different diseases are to be analyzed, including how the synchronization between these two types of signals changes.

## 6. Conclusions and Future Activity

The determined relative error in determining the number of QRS complexes and the average value of ECG and PPG time intervals in healthy individuals is small enough and we can say that the algorithm proposed by the authors to determine PP interval series can be used for correct PPG signal preprocessor processing.

The determined relative error in determining the same parameters in patients with syncope (for the examined ECG and PPG signals) is greater than 15%, which indicates impaired synchronization between ECG and PPG signals during their recording. 

Synchronization between ECG and PPG signals was impaired due to pathological changes in the cardiovascular system of the studied patients diagnosed with syncope. This may be another criterion for assessing the risk of cardiovascular disease.

The studies performed in the temporal domain show a good possibility to use the temporal parameters for the study of HRV in distinguishing patients with syncope from healthy individuals (parameters SDNN, SDANN and RMSSD have statistical significance, in which *p*-value < 0.005). All geometric measurements in the study have statistically significant results when comparing HRV in patients with syncope and healthy individuals. The conducted frequency analysis shows the possibility for effectively studying the signal strength in the low frequency range, in which significant differences were obtained between the values of the signal strength in patients diagnosed with syncope and healthy individuals. The sympathetic vagal balance index (LF/HF ratio) can also be used for diagnostic and prognostic purposes.

The research made in this paper shows the possibility of graphically distinguishing patients from healthy individuals. The applications of the two graphic methods using Histogram of PP interval series and Scaterogram of PP interval series show the presence of significant visual differences between the graphs of patients diagnosed with syncope and healthy individuals. The obtained graphical results show, in healthy individuals, a normal form of scattering (with the shape of a comet pointed at the bottom). A scaterogram in a patient with syncope shows torpedo-shaped scattering. This shows the advantage of using the graphical method for quick orientation in the health cardio status of the individual, while the numerical results presented in tabular form are studied more slowly but are more accurate and informative about the average values of all studied subjects included in the study.

The performed research studies are to be expanded by creating procedures for mathematical analysis of the two types of examined cardio signals with nonlinear methods, as well as with methods based on wavelet and fractal analysis for determination and research of numerical parameters. Other graphic methods for examination and graphical visualization of heart function using ECG and PPG will be applied. 

The research conducted in the present study, the results obtained, and the conclusions made provide the authors with reasons to undertake a study of HRV parameters (obtained using the PPG device and treated with the presented algorithm for PPG pretreatment) in patients with other heart diseases (e.g., myocardial infarction, ischemic heart disease, atrial fibrillation, rhythm and conduction disorders, condition of the body after significant heart disease, etc.). The authors are also working on creating an information database containing ECG and PPG records of patients with various heart diseases, and the records will be differentiated according to the type of diagnosis made by a cardiologist.

## Figures and Tables

**Table 1:** Table of demographic characteristics.

Parameters	Patients with Syncope *N* = 46	Healthy Subjects *N* = 48	*p*-Value
Gender, Men%	43.48	43.75	NS (0.1939)
Age ± sd	48.07 ± 6.14	45.85 ± 8.48	NS (0.1509)

**Table 2:** Table of defined parameters for the three types of signals for healthy individuals.

Parameters	ECG *N* = 20 (Mean ± Std)	PPG1 *N* = 20 (Mean ± Std)	PPG2 *N* = 20 (Mean ± Std)	Relative Error (%) ECG/PPG1	Relative Error (%) ECG/PPG2
Number of QRS complexes/pulse waves per 1 h	4976.73 ± 937.08	5103.65 ± 1080.57	5032.44 ± 988.12	2.49%	1.11%
Mean RR intervals/PP intervals (ms)	829.04 ± 152.43	851.11 ± 181.22	839.03 ± 164.86	2.59%	1.19%

**Table 3:** Table of defined parameters for the three types of signals for patients with syncope.

Parameters	ECG *N* = 20 (Mean ± Std)	PPG1 *N* = 20 (Mean ± Std)	PPG2 *N* = 20 (Mean ± Std)	Relative Error (%) ECG/PPG1	Relative Error (%) ECG/PPG2
Number of QRS complexes/pulse waves per 1 h	5358.12± 688.31	6309.71 ± 893.44	6313.71 ± 988.12	15.08%	15.14%
Mean RR intervals/PP intervals (ms)	734.09 ± 189.06	893.67 ± 181.22	902.54 ± 164.86	17.86%	18.66%

**Table 4:** Table for the determined parameters of analysis in the time and frequency domain.

Parameters		ECG *N* = 48 (Mean ± Std) Healthy Subjects	PPG *N* = 48 (Mean ± Std) Healthy Subjects	ECG *N* = 46 (Mean ± Std) Patients with Syncope	PPG *N* = 46 (Mean ± Std) Patients with Syncope	*p*-Value ECG Healthy/ Unhealthy	*p*-Value PPG Healthy/ Unhealthy
Time Domain	Statistical measurements						
	SDNN (ms)	142.61 ± 38.44	143.41 ± 39.48	86.89 ± 14.68	84.07 ± 22.03	<0.0001	<0.0001
	SDANN (ms)	146.07 ± 43.51	145.72 ± 46.73	91.86 ± 48.49	89.14 ± 49.56	<0.001 (0.0006)	<0.001 (0.0007)
	RMSSD (ms)	38.03 ± 12.09	37.83 ± 12.96	22.04 ± 19.11	24.72 ± 18.47	<0.005 (0.0031)	<0.05 (0.0133)
	SDindex (ms)	62.56 ± 30.76	63.44 ± 39.52	59.68 ± 34.73	60.11 ± 12.07	0.7828	0.7205
	Geometrical measurements						
	HRVti (number)	29.6 ± 5.5	29.3 ± 4.5	15 ± 2.1	18 ± 2.6	<0.0001	<0.0001
	TINN(ms)	288350.10 ± 921	278230.12 ± 890	501420.22 ± 1021	450150.03 ± 990	<0.0001	<0.0001
	SD1(ms)	35.04 ± 11	35.13 ± 9	20.03 ± 6	25.01 ± 4	<0.0001	<0.0001
	SD2(ms)	125.01 ± 22	124.07 ± 20	87.11 ± 16	61.06 ± 12	<0.0001	<0.0001
Frequency Domain	Power VLF $({\mathrm{ms}}^{2}$)	4176.1 ± 2097.25	4433.17 ± 2286.34	5603.76 ± 2946.23	5811.04 ± 2196.34	0.0855	0.0594
	Power LF $({\mathrm{ms}}^{2}$)	1873.35 ± 406.25	1806.22 ± 373.94	818.84 ± 232.06	831.09 ± 478.31	<0.0001	<0.0001
	Power HF $({\mathrm{ms}}^{2}$)	1110.42 ± 511.57	1042.31 ± 345.31	1297.22 ± 341.34	1189.98 ± 471.23	0.1824	0.2654
	Power VLF (%)	58.01 ± 28.38	60.88 ± 21.43	72.59 ± 39.03	74.20 ± 22.09	0.1846	0.0604
	Power LF (%)	26.45 ± 14.19	24.8 ± 15.09	10.6 ± 8.06	10.61 ± 9.08	<0.0001	<0.001 (0.0009)
	Power HF (%)	15.54 ± 8.07	14.31 ± 10.05	16.81 ± 6.27	15.18 ± 9.19	0.5816	0.7767
	LF/HF ratio	1.69 ± 0.76	1.73 ± 0.82	0.63 ± 0.41	0.69 ± 0.37	<0.0001	<0.0001

**Table 5:** Performance of parameters for Time-Domain and Frequency-Domain analysis of PPG data by using ROC statistics.

Parameters		AUC	Std. Error	Asymptotic 95% Confidence Interval	
				Lower Bound	Upper Bound
Time-Domain	SDNN (ms)	0.860	0.0936	0.633	0.972
	SDANN (ms)	0.880	0.0783	0.658	0.981
	RMSSD (ms)	0.850	0.0888	0.621	0.968
	SDindex (ms)	0.770	0.1160	0.530	0.926
	HRVti (number)	0.840	0.1060	0.609	0.963
	TINN(ms)	0.865	0.0916	0.639	0.975
	SD1(ms)	0.870	0.0880	0.645	0.977
	SD2(ms)	0.930	0.0550	0.723	0.996
Frequency-Domain	Power VLF (ms^2^)	0.770	0.1140	0.530	0.926
	Power LF (ms^2^)	0.920	0.0625	0.709	0.993
	Power HF (ms^2^)	0.635	0.1340	0.393	0.835
	Power VLF (%)	0.745	0.1200	0.504	0.910
	Power LF (%)	0.915	0.0663	0.703	0.992
	Power HF (%)	0.830	0.0923	0.598	0.958
	LF/HF ratio	0.945	0.0572	0.744	0.998

## Data Availability

The cardio data we processed for the research purposes of this paper were obtained from the Medical University of Varna, Bulgaria (available on http://hrvdata.vtlab.eu/, (accessed on 31 January 2022)).
